# Dynamics of invariant solutions of the DNA model using Lie symmetry approach

**DOI:** 10.1038/s41598-024-59983-8

**Published:** 2024-05-24

**Authors:** Akhtar Hussain, Muhammad Usman, Ahmed M. Zidan, Mohammed Sallah, Saud Owyed, Ariana Abdul Rahimzai

**Affiliations:** 1 Abdus Salam School of Mathematical Sciences, Government College University, 68-B New Muslim Town, Lahore, 54600 Pakistan; 2 College of Electrical and Mechanical Engineering (CEME), National University of Sciences and Technology (NUST), H-12, Islamabad, 44000 Pakistan; 3 Department of Mathematics, College of Science, King Khalid University, 61413 Abha, Saudi Arabia; 4 Applied Mathematical Physics Research Group, Physics Department, Faculty of Science, Mansoura University, Mansoura, 35516 Egypt; 5 Mathematics Department, College of Science, University of Bisha, P.O. Box 344, 61922 Bisha, Saudi Arabia; 6 Department of Mathematics, Education Faculty, Laghman University, Mehtarlam City, Laghman 2701 Afghanistan

**Keywords:** Lie group method, Mathematical physics, Microwave field, Symmetry algebra, Optical dark soliton, Engineering, Mathematics and computing, Optics and photonics, Physics

## Abstract

The utilization of the Lie group method serves to encapsulate a diverse array of wave structures. This method, established as a robust and reliable mathematical technique, is instrumental in deriving precise solutions for nonlinear partial differential equations (NPDEs) across a spectrum of domains. Its applications span various scientific disciplines, including mathematical physics, nonlinear dynamics, oceanography, engineering sciences, and several others. This research focuses specifically on the crucial molecule DNA and its interaction with an external microwave field. The Lie group method is employed to establish a five-dimensional symmetry algebra as the foundational element. Subsequently, similarity reductions are led by a system of one-dimensional subalgebras. Several invariant solutions as well as a spectrum of wave solutions is obtained by solving the resulting reduced ordinary differential equations (ODEs). These solutions govern the longitudinal displacement in DNA, shedding light on the characteristics of DNA as a significant real-world challenge. The interactions of DNA with an external microwave field manifest in various forms, including rational, exponential, trigonometric, hyperbolic, polynomial, and other functions. Mathematica simulations of these solutions confirm that longitudinal displacements in DNA can be expressed as periodic waves, optical dark solitons, singular solutions, exponential forms, and rational forms. This study is novel as it marks the first application of the Lie group method to explore the interaction of DNA molecules.

## Introduction

DNA stands as one of the most intricate and all-encompassing molecules in the realm of life. Numerous models aiming to describe the general properties of DNA dynamics prove to be intricate due to the multitude of elements inherent in each instance^1^.

The inaugural demonstration of resonant microwave absorption in DNA was conducted by Webb and Booth^2^. Subsequent investigations into the microwave absorption characteristics of DNA were undertaken by Swicord and Davis^3,4^. Nonetheless, the outcomes reported by Gabriel et al.^5^, Yakushevich^6^, Bixon et al.^7^, Henderson^8^, and Bruinsma^9^ have introduced a degree of controversy to these observations. Consequently, diverse methodologies have been proposed to articulate models of DNA. Yakushevich^6^ extensively delved into the nonlinear properties inherent in the physics of DNA. Some DNA models have been predicated on linear constructs^5–11^, whereas others have embraced nonlinear frameworks^12–14^. Muto et al. were pioneers in presenting a nonlinear mathematical model elucidating the interaction between DNA and an external microwave field^15^

1 $$\begin{aligned} u_{tt}-\alpha ^{2}u_{zz}+\frac{\vartheta _3}{\alpha ^{2}}u_{zztt}-\gamma (u_{z}^{2})_z =0, \end{aligned}$$

the notation *u*(*z*, *t*) is employed to characterize longitudinal displacements in DNA^12,13^. Deciphering the concealed characteristics of DNA poses a significant real-world challenge. Recently, Kong et al.^1^, Alka et al.^15^, and Abdelrahman et al.^16^ have proposed an innovative physical-mathematical model for double-chain DNA. This model envisions DNA as comprising two extended, elastic, homogeneous strands connected by an elastic membrane, symbolizing the hydrogen bonds between the base pairs of the two chains.

The Lie group method^17,18^ stands out as a fundamental and potent tool in addressing various aspects such as invariant solutions, conservation laws, linearization, reducing the order of nonlinearity in nonlinear problems, and assessing the stability of a numerical scheme. Pioneered by Sophus Lie and notably advanced by Ovsiannikov^19^, Ibragimov^20^, Bluman^21^, Olver^22^, and others, this method has found applications in diverse problem domains. It has been successfully applied to challenges ranging from nonlinear elastic structural element equations^23^ to the beam equation in the Timoshenko model^24^, the (3+1)-dimensional generalized nonlinear evolution equation in shallow water waves^25^, the Slepyan-Palmov Model in the Slepyan-Palmov Medium^26^, and the Thomas equation using symmetry transformations^27^. The method has also been extended to discrete domain equations^28^.

In this context, our motivation is to employ this powerful method to explore the characteristics of displacement in DNA and its interactions with an external microwave field. By applying the Lie group method^29–38^ to the study of DNA molecules, we can leverage the group structure to elucidate a broad class of wave spectrum. This spectrum provides insights into the nature of DNA displacement, expressing it as periodic waves, optical dark solitons, singular solutions, exponential forms, and rational forms. These results are groundbreaking and represent novel contributions not previously documented in the theory of DNA molecules.

The structure of the paper unfolds as follows: In Sect. "Invariant analysis and the optimal subalgebraic system", we delve into applying the Lie group method to the DNA Eq. (1) and explore its optimal system. Section "Invariant solutions via non similar classes" employs the optimal system to derive invariant solutions and reduced ODEs. The new auxiliary equation method is introduced in Sect. "The new auxiliary equation method", and its implementation to the DNA Eq. (1) is detailed in Sect. "Implementation of new auxiliary equation method". Section "Physical nature of the obtained solutions" provides an overview of the nature of longitudinal displacement in DNA based on the solutions obtained. The paper concludes in Sect. "Discussion and conclusions", offering a summary and pointing towards potential future directions.

## Invariant analysis and the optimal subalgebraic system

This section is dedicated to the comprehensive analysis of Lie symmetries and the optimal system corresponding to Eq. (1). We initiate our investigation by considering a one-parameter Lie group of transformations^22^

2 $$\begin{aligned} \begin{aligned} \tilde{z}\rightarrow z+\varepsilon \phi _{1}(z,t,u)+O(\varepsilon ^{2}),\\ \tilde{t}\rightarrow t+\varepsilon \phi _{2}(z,t,u)+O(\varepsilon ^{2}),\\ \tilde{u}\rightarrow u+\varepsilon \vartheta (z,t,u)+O(\varepsilon ^{2}), \end{aligned} \end{aligned}$$

where $\varepsilon$ is the parameter of a Lie group. The transformations mentioned above have an associated infinitesimal generator

3 $$\begin{aligned} {\mathcal {Y}}=\phi _{1}(z,t,u)\frac{\partial }{\partial z}+\phi _{2}(z,t,u)\frac{\partial }{\partial t}+\vartheta (z,t,u) \frac{\partial }{\partial u}\cdot \end{aligned}$$

The central aim is to identify the coefficient functions $\phi _{1}, \phi _{2}$, and $\vartheta$, while verifying that the operator ${\mathcal {Y}}$ conforms to the requirements of the Lie symmetry condition

4 $$\begin{aligned} {\mathcal {Y}}^{[4]}(\Delta )|_{\Delta =0}=0, \end{aligned}$$

where ${\mathcal {Y}}^{[4]}$ denotes the fourth prolongation of ${\mathcal {Y}}$ and

$$\begin{aligned} \Delta =u_{tt}-\alpha ^{2}u_{zz}+\frac{\vartheta _3}{\alpha ^{2}}u_{zztt}-\gamma (u_{z}^{2})_z. \end{aligned}$$

Through the resolution of Eq. (4), the infinitesimal terms are determined and can be expressed as,

$$\begin{aligned} \phi _{1}=c_3,\quad \phi _{2}=c_1 t+c_2,\quad \vartheta =-2c_1 u-\frac{c_1}{b}\alpha ^{2}z+c_4 t+c_5, \end{aligned}$$

which leads to the five-dimensional Lie algebra of Eq. (1) given by

5 $$\begin{aligned} {\mathcal {Y}}_{1}=\frac{\partial }{\partial t},\quad {\mathcal {Y}}_{2}=\frac{\partial }{\partial u},\quad {\mathcal {Y}}_{3}=\frac{\partial }{\partial z},\quad {\mathcal {Y}}_4 =t\frac{\partial }{\partial u},\quad {\mathcal {Y}}_5 =t\frac{\partial }{\partial t}+(-2u-\frac{\alpha ^{2}}{\gamma }z)\frac{\partial }{\partial u}\cdot \end{aligned}$$

**Table 1:** Commutator table.

$[{\mathcal {Y}}_{m},{\mathcal {Y}}_{n}]$	${\mathcal {Y}}_{1}$	${\mathcal {Y}}_{2}$	${\mathcal {Y}}_{3}$	${\mathcal {Y}}_{4}$	${\mathcal {Y}}_{5}$
${\mathcal {Y}}_{1}$	0	0	0	${\mathcal {Y}}_2$	${\mathcal {Y}}_1$
${\mathcal {Y}}_{2}$	0	0	0	0	$-2{\mathcal {Y}}_2$
${\mathcal {Y}}_{3}$	0	0	0	0	$-\frac{\alpha ^{2}}{\gamma }{\mathcal {Y}}_2$
${\mathcal {Y}}_{4}$	$-{\mathcal {Y}}_2$	0	0	0	$-3{\mathcal {Y}}_4$
${\mathcal {Y}}_{5}$	$-{\mathcal {Y}}_1$	$2{\mathcal {Y}}_2$	$\frac{\alpha ^{2}}{\gamma }{\mathcal {Y}}_2$	$3{\mathcal {Y}}_4$	0

We can write down the representation of the adjoint action as (Table 1),

6 $$\begin{aligned} Ad(\exp {(\varepsilon {\mathcal {Y}}_m )}.{\mathcal {Y}}_n) = {\mathcal {Y}}_n -\varepsilon [{\mathcal {Y}}_m, {\mathcal {Y}}_n ]+\frac{\varepsilon ^{2}}{2!}[{\mathcal {Y}}_m,[{\mathcal {Y}}_m, {\mathcal {Y}}_n ]]-\cdots . \end{aligned}$$

By utilizing the adjoint expression (6), we can create the adjoint representation table, which is provided in Table 2.

**Table 2:** Adjoint table.

$Ad(e^{\varepsilon })$	${\mathcal {Y}}_{1}$	${\mathcal {Y}}_{2}$	${\mathcal {Y}}_{3}$	${\mathcal {Y}}_{4}$	${\mathcal {Y}}_{5}$
${\mathcal {Y}}_{1}$	${\mathcal {Y}}_1$	${\mathcal {Y}}_2$	${\mathcal {Y}}_3$	${\mathcal {Y}}_4 -\varepsilon {\mathcal {Y}}_2$	${\mathcal {Y}}_5 -\varepsilon {\mathcal {Y}}_1$
${\mathcal {Y}}_{2}$	${\mathcal {Y}}_1$	${\mathcal {Y}}_2$	${\mathcal {Y}}_3$	${\mathcal {Y}}_4$	${\mathcal {Y}}_5 +2\varepsilon {\mathcal {Y}}_2$
${\mathcal {Y}}_{3}$	${\mathcal {Y}}_1$	${\mathcal {Y}}_2$	${\mathcal {Y}}_3$	${\mathcal {Y}}_4$	${\mathcal {Y}}_5 +\frac{\alpha ^{2}}{\gamma }\varepsilon {\mathcal {Y}}_2$
${\mathcal {Y}}_{4}$	${\mathcal {Y}}_1 +\varepsilon {\mathcal {Y}}_2$	${\mathcal {Y}}_2$	${\mathcal {Y}}_3$	${\mathcal {Y}}_4$	${\mathcal {Y}}_5 +3\varepsilon {\mathcal {Y}}_4$
${\mathcal {Y}}_{5}$	$e^{\varepsilon }{\mathcal {Y}}_1$	$e^{-2\varepsilon }{\mathcal {Y}}_2$	${\mathcal {Y}}_3 +\frac{\alpha ^{2}}{2\gamma }(-1+e^{-2\varepsilon }){\mathcal {Y}}_2$	$e^{-3\varepsilon } {\mathcal {Y}}_4$	${\mathcal {Y}}_5$

### Optimal system

Consider an arbitrary element ${\mathcal {Y}}$ of five-dimensional Lie algebra $\theta ^{5}$ given by,

7 $$\begin{aligned} {\mathcal {Y}}=k_1 {\mathcal {Y}}_1 +k_2 {\mathcal {Y}}_2 +k_3 {\mathcal {Y}}_3 +k_4 {\mathcal {Y}}_4 +k_5 {\mathcal {Y}}_5. \end{aligned}$$

We will employ the adjoint action provided in Table 2 to simplify the coefficients in (7) as extensively as possible.
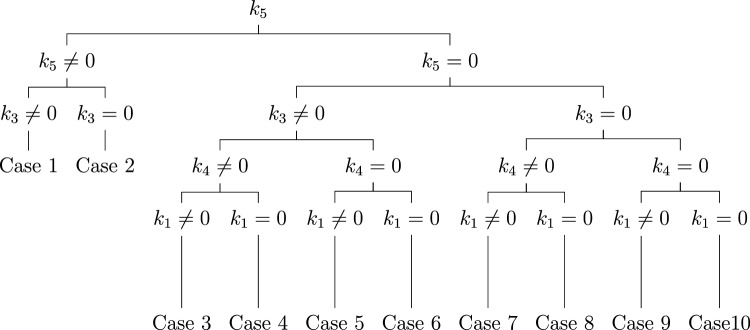


$${\text {Case 1}}$$: $$k_5 \ne 0,k_3 \ne 0$$, then (7) becomes

8 $$\begin{aligned} {\mathcal {Y}}= & {} k_1 {\mathcal {Y}}_1 +k_2 {\mathcal {Y}}_2 +k_3 {\mathcal {Y}}_3 +k_4 {\mathcal {Y}}_4 +k_5 {\mathcal {Y}}_5 \end{aligned}$$

9 $$\begin{aligned} {\mathcal {Y}}'= & {} Ad(e^{\varepsilon }{\mathcal {Y}}_1){\mathcal {Y}}=k_3 {\mathcal {Y}}_3 +k_4 {\mathcal {Y}}_4 +k_5 {\mathcal {Y}}_5 \end{aligned}$$

10 $$\begin{aligned} {\mathcal {Y}}''= & {} Ad(e^{\varepsilon }{\mathcal {Y}}_4){\mathcal {Y}}'=k_3 {\mathcal {Y}}_3 +k_5 {\mathcal {Y}}_5 \end{aligned}$$

By taking, $k_3 =1$, we obtain,

11 $$\begin{aligned} \Lambda _1 ={\mathcal {Y}}_3 +c{\mathcal {Y}}_5,~c\ne 0. \end{aligned}$$

$${\text {Case 2}}$$: $$k_5 \ne 0,k_3 = 0$$, then (7) becomes

12 $$\begin{aligned} {\mathcal {Y}}= & {} k_1 {\mathcal {Y}}_1 +k_2 {\mathcal {Y}}_2 +k_4 {\mathcal {Y}}_4 +k_5 {\mathcal {Y}}_5 \end{aligned}$$

13 $$\begin{aligned} {\mathcal {Y}}'= & {} Ad(e^{\varepsilon }{\mathcal {Y}}_1){\mathcal {Y}}=k_4 {\mathcal {Y}}_4 +k_5 {\mathcal {Y}}_5 \end{aligned}$$

14 $$\begin{aligned} {\mathcal {Y}}''= & {} Ad(e^{\varepsilon }{\mathcal {Y}}_4){\mathcal {Y}}'=k_5 {\mathcal {Y}}_5 \end{aligned}$$

So, we obtain,

15 $$\begin{aligned} \Lambda _2 ={\mathcal {Y}}_5. \end{aligned}$$

$${\text {Case 3}}$$: $$k_5 = 0,k_4 \ne 0,k_3 \ne 0, k_1 \ne 0$$, then (7) becomes

16 $$\begin{aligned} {\mathcal {Y}}= & {} k_1 {\mathcal {Y}}_1 +k_2 {\mathcal {Y}}_2 +k_3 {\mathcal {Y}}_3 +k_4 {\mathcal {Y}}_4 \end{aligned}$$

17 $$\begin{aligned} {\mathcal {Y}}'= & {} Ad(e^{\varepsilon }{\mathcal {Y}}_5){\mathcal {Y}}=k_1 {\mathcal {Y}}_1 +e^{-\varepsilon } k_2 {\mathcal {Y}}_2 +e^{-4\varepsilon }k_3 {\mathcal {Y}}_3 \end{aligned}$$

By taking $k_1 =1$, we get,

18 $$\begin{aligned} \Lambda _3 ={\mathcal {Y}}_1 \pm {\mathcal {Y}}_3 \pm {\mathcal {Y}}_4. \end{aligned}$$

$${\text {Case 4}}$$: $$k_5 = 0,k_4 \ne 0,k_3 \ne 0, k_1 = 0$$, then (7) becomes,

19 $$\begin{aligned} {\mathcal {Y}}= & {} k_2 {\mathcal {Y}}_2 +k_3 {\mathcal {Y}}_3 +k_4 {\mathcal {Y}}_4 \end{aligned}$$

20 $$\begin{aligned} {\mathcal {Y}}'= & {} Ad(e^{\varepsilon }{\mathcal {Y}}_1){\mathcal {Y}}= k_3 {\mathcal {Y}}_3 +k_4 {\mathcal {Y}}_4 \end{aligned}$$

21 $$\begin{aligned} {\mathcal {Y}}''= & {} Ad(e^{\varepsilon }{\mathcal {Y}}_5 ){\mathcal {Y}}'= k_3 {\mathcal {Y}}_3 +e^{-3\varepsilon }k_4 {\mathcal {Y}}_4 \end{aligned}$$

By taking $k_3 =1$, we get,

22 $$\begin{aligned} \Lambda _4 = {\mathcal {Y}}_3 \pm {\mathcal {Y}}_4. \end{aligned}$$

$${\text {Case 5}}$$: $$k_5 = 0,k_4 = 0,k_3 \ne 0, k_1 \ne 0$$, then (7) becomes

23 $$\begin{aligned} {\mathcal {Y}}= & {} k_1 {\mathcal {Y}}_1 +k_2 {\mathcal {Y}}_2 +k_3 {\mathcal {Y}}_3 \end{aligned}$$

24 $$\begin{aligned} {\mathcal {Y}}'= & {} Ad(e^{\varepsilon }{\mathcal {Y}}_4){\mathcal {Y}}= k_1 {\mathcal {Y}}_1 +k_3 {\mathcal {Y}}_3 \end{aligned}$$

25 $$\begin{aligned} {\mathcal {Y}}''= & {} Ad(e^{\varepsilon }{\mathcal {Y}}_5 ){\mathcal {Y}}'= k_1 {\mathcal {Y}}_1 +e^{-\varepsilon }k_3 {\mathcal {Y}}_3 \end{aligned}$$

By taking, $k_1 =1$, we get,

26 $$\begin{aligned} \Lambda _5 = {\mathcal {Y}}_1 \pm {\mathcal {Y}}_3. \end{aligned}$$

$${\text {Case 6}}$$: $$k_5 = 0,k_4 = 0,k_3 \ne 0, k_1 = 0$$, then (7) becomes

27 $$\begin{aligned} {\mathcal {Y}}= & {} k_2 {\mathcal {Y}}_2 +k_3 {\mathcal {Y}}_3 \end{aligned}$$

28 $$\begin{aligned} {\mathcal {Y}}'= & {} Ad(e^{\varepsilon }{\mathcal {Y}}_5){\mathcal {Y}}=k_3 {\mathcal {Y}}_3 \end{aligned}$$

So, we get,

29 $$\begin{aligned} \Lambda _6 = {\mathcal {Y}}_3. \end{aligned}$$

$${\text {Case 7}}$$: $$k_5 = 0,k_4 \ne 0,k_3 = 0, k_1 \ne 0$$, then (7) becomes

30 $$\begin{aligned} {\mathcal {Y}}= & {} k_1 {\mathcal {Y}}_1 +k_2 {\mathcal {Y}}_2 +k_4 {\mathcal {Y}}_4 \end{aligned}$$

31 $$\begin{aligned} {\mathcal {Y}}'= & {} Ad(e^{\varepsilon }{\mathcal {Y}}_1){\mathcal {Y}}=k_1 {\mathcal {Y}}_1 +k_4 {\mathcal {Y}}_4 \end{aligned}$$

32 $$\begin{aligned} {\mathcal {Y}}''= & {} Ad(e^{\varepsilon }{\mathcal {Y}}_5){\mathcal {Y}}'=k_1 {\mathcal {Y}}_1 +e^{-4\varepsilon } k_4 {\mathcal {Y}}_4 \end{aligned}$$

By taking $k_1 =1$, we obtain,

33 $$\begin{aligned} \Lambda _7 = {\mathcal {Y}}_1 \pm {\mathcal {Y}}_4. \end{aligned}$$

$${\text {Case 8}}$$: $$k_5 = 0,k_4 \ne 0,k_3 = 0, k_1 = 0$$, then (7) becomes,

34 $$\begin{aligned} {\mathcal {Y}}= & {} k_2 {\mathcal {Y}}_2 +k_4 {\mathcal {Y}}_4 \end{aligned}$$

35 $$\begin{aligned} {\mathcal {Y}}'= & {} Ad(e^{\varepsilon }{\mathcal {Y}}_1){\mathcal {Y}}=k_4 {\mathcal {Y}}_4 \end{aligned}$$

So, we obtain,

36 $$\begin{aligned} \Lambda _8 = {\mathcal {Y}}_4. \end{aligned}$$

$${\text {Case 9}}$$: $$k_5 = 0,k_4 = 0,k_3 = 0, k_1 \ne 0$$, then (7) becomes,

37 $$\begin{aligned} {\mathcal {Y}}= & {} k_1 {\mathcal {Y}}_1 +k_2 {\mathcal {Y}}_2 \end{aligned}$$

38 $$\begin{aligned} {\mathcal {Y}}'= & {} Ad(e^{\varepsilon }{\mathcal {Y}}_4){\mathcal {Y}}=k_1 {\mathcal {Y}}_1 \end{aligned}$$

So, we obtain,

39 $$\begin{aligned} \Lambda _9 = {\mathcal {Y}}_1. \end{aligned}$$

$${\text {Case 10}}$$: $$k_5 = 0,k_4 = 0,k_3 = 0, k_1 =0$$, then (7) becomes

40 $$\begin{aligned} {\mathcal {Y}}= k_2 {\mathcal {Y}}_2 \end{aligned}$$

So, we get,

41 $$\begin{aligned} \Lambda _{10} = {\mathcal {Y}}_2. \end{aligned}$$

Accordingly, the one-dimensional optimal organization for Lie algebra (5) is detailed as

42 $$\begin{aligned} \begin{aligned} \Lambda _1&={\mathcal {Y}}_3 +c{\mathcal {Y}}_5,~c\ne 0,\\ \Lambda _2&={\mathcal {Y}}_5,\\ \Lambda _3&={\mathcal {Y}}_1 \pm {\mathcal {Y}}_3 \pm {\mathcal {Y}}_4,\\ \Lambda _4&={\mathcal {Y}}_3 \pm {\mathcal {Y}}_4,\\ \Lambda _5&={\mathcal {Y}}_1 \pm {\mathcal {Y}}_3,\\ \Lambda _6&={\mathcal {Y}}_3,\\ \Lambda _7&={\mathcal {Y}}_1 \pm {\mathcal {Y}}_4,\\ \Lambda _8&={\mathcal {Y}}_4,\\ \Lambda _9&={\mathcal {Y}}_1,\\ \Lambda _{10}&={\mathcal {Y}}_2, \end{aligned} \end{aligned}$$

where two real parameters, denoted as *c* and *d* in the given context, consistently maintain a non-zero status.

## Invariant solutions via non similar classes

Within this section, we introduce invariant solutions that are explicitly formulated after subjecting the system to symmetry reduction under the optimal configuration (42). Employing similarity reductions, the nonlinear Eq. (1) undergoes simplification, transforming into ordinary differential equations (ODEs) recognized as similarity reduction equations. These equations possess the capability to produce solutions that exhibit invariance under group transformations.

### Invariant solution by non similar class

$$\Lambda _{9}=\langle {\mathcal {Y}}_{1} \rangle $$.

Taking into account the symmetry generator, ${\mathcal {Y}}_{1}=\frac{\partial }{\partial t}$, the characteristic equation is presented as follows:

$$\begin{aligned} \frac{dz}{0}=\frac{dt}{1}=\frac{du}{0}\cdot \end{aligned}$$

The use of similarity variables, $u = h(\sigma )$ and $\sigma = z$ leads to the Eq. (1) being reduced to an ordinary differential equation,

43 $$\begin{aligned} -h''(2\gamma h' +\alpha ^{2})=0. \end{aligned}$$

If $h''=0$, this gives $h(\sigma )=c_1 \sigma +c_2.$ So, the exact solution of (1) becomes

44 $$\begin{aligned} u(z,t)=c_1 z+c_2. \end{aligned}$$

If $h'' \ne 0,$ then $2\gamma h' +\alpha ^{2} =0$, which yields $h(\sigma )=c_1 -\frac{\alpha ^{2}}{2\gamma } \sigma .$ Thus, the invariant solution for the DNA Eq. (1) is written as,

45 $$\begin{aligned} u(z,t)=c_1 -\frac{\alpha ^{2}}{2\gamma } z. \end{aligned}$$

### Invariant solution by non similar class

$$\Lambda _{6}=\langle {\mathcal {Y}}_{3} \rangle $$.

Taking into account the symmetry generator ${\mathcal {Y}}_{3}=\frac{\partial }{\partial z}$, the characteristic equation is presented as follows

$$\begin{aligned} \frac{dz}{1}=\frac{dt}{0}=\frac{du}{0}\cdot \end{aligned}$$

The use of similarity variables $u = h(\sigma )$ and $\sigma = t$ leads to the Eq. (1) being reduced to an ordinary differential equation

46 $$\begin{aligned} h''=0, \end{aligned}$$

this gives,

47 $$\begin{aligned} h(\sigma )=c_1 \sigma +c_2. \end{aligned}$$

Thus, the invariant solution for the DNA Eq. (1) is written as

48 $$\begin{aligned} u(z,t)=c_1 t+c_2. \end{aligned}$$

### Invariant solution by non similar class

$$\Lambda _{7}=\langle {\mathcal {Y}}_{1} +{\mathcal {Y}}_4 \rangle $$.

Taking into account the symmetry generator ${\mathcal {Y}}_{1}+{\mathcal {Y}}_4 =\frac{\partial }{\partial t}+t\frac{\partial }{\partial u}$, the characteristic equation is presented as follows

$$\begin{aligned} \frac{dz}{0}=\frac{dt}{1}=\frac{du}{t}\cdot \end{aligned}$$

The use of similarity variables $u = \frac{t^{2}}{2}+h(\sigma )$ and $\sigma = z$ leads to the Eq. (1) being reduced to an ordinary differential equation

49 $$\begin{aligned} -2\gamma h' h'' -\alpha ^{2}h''+1=0. \end{aligned}$$

This gives,

50 $$\begin{aligned} h(\sigma )=\frac{-6\alpha ^{2}\gamma \sigma +((4c_1 +4\sigma ) \gamma +\alpha ^{4})^{\frac{3}{2}}}{12\gamma ^{2}}+c_2\cdot \end{aligned}$$

Thus, the invariant solution for the DNA Eq. (1) is written as

51 $$\begin{aligned} u(z,t)=\frac{(6t^{2}+12c_2 )\gamma ^{2}-6\alpha ^{2}\gamma z +((4c_1 +4z) \gamma +\alpha ^{4})^{\frac{3}{2}}}{12\gamma ^{2}}\cdot \end{aligned}$$

### Invariant solution by non similar class

$$\Lambda _{7}=\langle {\mathcal {Y}}_{1} -{\mathcal {Y}}_4 \rangle $$.

Taking into account the symmetry generator ${\mathcal {Y}}_{1}-{\mathcal {Y}}_4 =\frac{\partial }{\partial t}-t\frac{\partial }{\partial u}$, the characteristic equation is presented as follows

$$\begin{aligned} \frac{dz}{0}=\frac{dt}{1}=\frac{du}{-t}\cdot \end{aligned}$$

The use of similarity variables $u = -\frac{t^{2}}{2}+h(\sigma )$ and $\sigma = z$ leads to the Eq. (1) being reduced to an ordinary differential equation

52 $$\begin{aligned} 2\gamma h' h'' +\alpha ^{2}h''+1=0, \end{aligned}$$

this gives,

53 $$\begin{aligned} h(\sigma )=\frac{-6\alpha ^{2}\gamma \sigma +((-4c_1 -4\sigma ) \gamma +\alpha ^{4})^{\frac{3}{2}}}{12\gamma ^{2}}+c_2. \end{aligned}$$

Thus, the invariant solution for the DNA Eq. (1) is written as

54 $$\begin{aligned} u(z,t)=\frac{(-6t^{2}+12c_2 )\gamma ^{2}-6\alpha ^{2}\gamma z +((-4c_1 -4z) \gamma +\alpha ^{4})^{\frac{3}{2}}}{12\gamma ^{2}} \cdot \end{aligned}$$

### Invariant solution by non similar class

$$\Lambda _{4}=\langle {\mathcal {Y}}_{3} +{\mathcal {Y}}_4 \rangle $$.

Taking into account the symmetry generator ${\mathcal {Y}}_{3}+{\mathcal {Y}}_4 =\frac{\partial }{\partial z}+t\frac{\partial }{\partial u}$, the characteristic equation is presented as follows

$$\begin{aligned} \frac{dz}{1}=\frac{dt}{0}=\frac{du}{t}\cdot \end{aligned}$$

The use of similarity variables $u = zt+h(\sigma )$ and $\sigma = t$ leads to the Eq. (1) being reduced to an ordinary differential equation

55 $$\begin{aligned} h'' =0, \end{aligned}$$

this gives,

56 $$\begin{aligned} h(\sigma )=c_1 \sigma +c_2. \end{aligned}$$

Thus, the invariant solution for the DNA Eq. (1) is written as

57 $$\begin{aligned} u(z,t)=(c_1 +z)t+c_2. \end{aligned}$$

### Invariant solution by non similar class

$$\Lambda _{4}=\langle {\mathcal {Y}}_{3} -{\mathcal {Y}}_4 \rangle $$.

Taking into account the symmetry generator ${\mathcal {Y}}_{3}-{\mathcal {Y}}_4 =\frac{\partial }{\partial z}-t\frac{\partial }{\partial u}$, the characteristic equation is presented as follows

$$\begin{aligned} \frac{dz}{1}=\frac{dt}{0}=\frac{du}{-t}\cdot \end{aligned}$$

The use of similarity variables $u = -zt+h(\sigma )$ and $\sigma = t$ leads to the Eq. (1) being reduced to an ordinary differential equation

58 $$\begin{aligned} h'' =0, \end{aligned}$$

this gives,

59 $$\begin{aligned} h(\sigma )=c_1 \sigma +c_2. \end{aligned}$$

Thus, the invariant solution for the DNA Eq. (1) is written as

60 $$\begin{aligned} u(z,t)=(c_1 -z)t+c_2. \end{aligned}$$

### Invariant solution by non similar class

$$\Lambda _{2}=\langle {\mathcal {Y}}_{5} \rangle $$.

Taking into account the symmetry generator ${\mathcal {Y}}_{5} =t\frac{\partial }{\partial t}+(-2u-\frac{\alpha ^{2}}{\gamma }z)\frac{\partial }{\partial u}$, the characteristic equation is presented as follows

$$\begin{aligned} \frac{dz}{0}=\frac{dt}{t}=\frac{du}{(-2u-\frac{\alpha ^{2}}{\gamma }z)}\cdot \end{aligned}$$

The use of similarity variables $u = \frac{-\alpha ^{2}zt^{2}+2\gamma h(\sigma )}{2\gamma t^{2}}$ and $\sigma = z$ leads to the Eq. (1) being reduced to an ordinary differential equation

61 $$\begin{aligned} (-2\alpha ^{2}\gamma h' +6\vartheta _3 )h'' +6\alpha ^{2}h =0. \end{aligned}$$

We propose solving the aforementioned ODE numerically.

### Invariant solution by non similar class

$$\Lambda _{5}=\langle {\mathcal {Y}}_{1} +{\mathcal {Y}}_3 \rangle $$.

Taking into account the symmetry generator ${\mathcal {Y}}_{1}+{\mathcal {Y}}_3 =\frac{\partial }{\partial t}+\frac{\partial }{\partial z}$, the characteristic equation is presented as follows

$$\begin{aligned} \frac{dz}{1}=\frac{dt}{1}=\frac{du}{0}\cdot \end{aligned}$$

The use of similarity variables $u = h(\sigma )$ and $\sigma = t-z$ leads to the Eq. (1) being reduced to an ordinary differential equation

62 $$\begin{aligned} -\alpha ^{2}(\alpha ^{2}-2\gamma h' -1)h'' +\vartheta _3 h^{(iv)} =0. \end{aligned}$$

### Invariant solution by non similar class

$$\Lambda _{3}=\langle {\mathcal {Y}}_{1} +{\mathcal {Y}}_3 +{\mathcal {Y}}_4 \rangle $$.

Taking into account the symmetry generator ${\mathcal {Y}}_{1}+{\mathcal {Y}}_3 +{\mathcal {Y}}_4 =\frac{\partial }{\partial t}+\frac{\partial }{\partial z}+t\frac{\partial }{\partial u}$, the characteristic equation is presented as follows

$$\begin{aligned} \frac{dz}{1}=\frac{dt}{1}=\frac{du}{t}\cdot \end{aligned}$$

The use of similarity variables $u = -\frac{z^{2}}{2}+zt+h(\sigma )$ and $\sigma = -z+t$ leads to the Eq. (1) being reduced to an ordinary differential equation

63 $$\begin{aligned} \vartheta _3 h^{(iv)} -2\alpha ^{2}\Big ((\gamma \sigma +\frac{\alpha ^{2}}{2}-\gamma h'-\frac{1}{2})h''-\gamma \sigma -\frac{\alpha ^{2}}{2}+\gamma h'\Big )=0. \end{aligned}$$

We propose solving the aforementioned ODE numerically.

## The new auxiliary equation method

Consider a general nonlinear partial differential equation (PDE) represented as

64 $$\begin{aligned} Q(u,u_z,u_t, u_{zz},\cdots )=0, \end{aligned}$$

where *Q* is a polynomial function of *u* and their derivatives with respect to two independent variables *z* and *t*. The procedure has a few phases, which are listed below;

*Step: 1* Suppose a new dependent and an independent variable as

65 $$\begin{aligned} u(z,t)=h(\sigma ),\qquad \sigma =z-ct, \end{aligned}$$

where $\sigma$ is a new independent variable, with *c* representing a real parameter for Eq. (64). By substituting Eq. (65) into Eq. (64), we obtain the following ODE;

66 $$\begin{aligned} {\mathcal {P}}(h,h^\prime ,h^{\prime \prime },...)=0. \end{aligned}$$

*Step: 2* Consider a solution for Eq. (66) in the following form

67 $$\begin{aligned} h(\sigma )=\sum _{i=0}^{k}b_i\Theta ^{ih(\sigma )}, \end{aligned}$$

which satisfies the auxiliary equation

68 $$\begin{aligned} h^\prime (\sigma )=\frac{1}{\ln (\Theta )}\{\Gamma _1+\Gamma _2 \Theta ^{h(\sigma )}+\Gamma _3 \Theta ^{-h(\sigma )}\},~~\Theta >0,~~\Theta \ne 1, \end{aligned}$$

where $b_{i}'s$ are constants which will be computed later.

*Step: 3* To determine the value of *k* in Eq. (67), we employ the balancing procedure, where we compare the highest-order nonlinear term with the highest-order derivative.

*Step: 4* By substituting Eqs. (67) and (68) into Eq. (66) and collecting the coefficients of various powers of $\Theta ^{h(\sigma )}$ $(i=0,1,2,\cdots )$, we form a system of equations. Setting all coefficients equal to zero yields a system that can be solved using Maple software to obtain the solution.

*Step: 5* The nature of solutions for Eq. (68) can be determined as;

*Case:1* When $\vartheta ^2_1-\vartheta _2\vartheta _3<0$ and $\vartheta _3\ne 0$

69 $$\begin{aligned} \Theta ^{h(\sigma )}= & {} \frac{-\vartheta _1}{\vartheta _3}+\frac{\sqrt{-(\vartheta _1^2-\vartheta _2\vartheta _3)}}{\vartheta _3}\tan \bigg (\frac{\sqrt{-(\vartheta _1 ^2-\vartheta _2\vartheta _3)}}{2}\sigma \bigg ), \end{aligned}$$

70 $$\begin{aligned} \Theta ^{h(\sigma )}= & {} \frac{-\vartheta _1}{\vartheta _3}+\frac{\sqrt{-(\vartheta _1^2-\vartheta _2\vartheta _3)}}{\vartheta _3}\cot \bigg (\frac{\sqrt{-(\vartheta _1 ^2-\vartheta _2\vartheta _3)}}{2}\sigma \bigg ). \end{aligned}$$

*Case:2* When $\vartheta _1^2+\vartheta _2\vartheta _3>0$ and $\vartheta _3\ne 0$

71 $$\begin{aligned} \Theta ^{h(\sigma )}= & {} \frac{-\vartheta _1}{\vartheta _3}+\frac{\sqrt{(\vartheta _1^2-\vartheta _2\vartheta _3)}}{\vartheta _3}\tanh \bigg (\frac{\sqrt{(\vartheta _1 ^2-\vartheta _2\vartheta _3)}}{2}\sigma \bigg ), \end{aligned}$$

72 $$\begin{aligned} \Theta ^{h(\sigma )}= & {} \frac{-\vartheta _1}{\vartheta _3}-\frac{\sqrt{(\vartheta _1^2-\vartheta _2\vartheta _3)}}{\vartheta _3}\coth \bigg (\frac{\sqrt{(\vartheta _1 ^2-\vartheta _2\vartheta _3)}}{2}\sigma \bigg ). \end{aligned}$$

*Case:3* When $\vartheta _1^2+\vartheta _2\vartheta _3>0$ and $\vartheta _3\ne 0$ and $\vartheta _3\ne -\vartheta _2$

73 $$\begin{aligned} \Theta ^{h(\sigma )}= & {} \frac{\vartheta _1}{\vartheta _3}+\frac{\sqrt{(\vartheta _1^2+\vartheta _2^2)}}{\vartheta _3}\tanh \bigg (\frac{\sqrt{(\vartheta _1^2+\vartheta _2^2)}}{2}\sigma \bigg ), \end{aligned}$$

74 $$\begin{aligned} \Theta ^{h(\sigma )}= & {} \frac{\vartheta _1}{\vartheta _3}+\frac{\sqrt{(\vartheta _1^2+\vartheta _2^2)}}{\vartheta _3}\coth \bigg (\frac{\sqrt{(\vartheta _1^2+\vartheta _2^2)}}{2}\sigma \bigg ). \end{aligned}$$

*Case: 4* When $\vartheta _1^2+\vartheta _2\vartheta _3<0$, $\vartheta _3\ne 0$ and $\vartheta _3\ne -\vartheta _2$

75 $$\begin{aligned} \Theta ^{h(\sigma )}= & {} \frac{\vartheta _1}{\vartheta _3}+\frac{\sqrt{-(\vartheta _1^2+\vartheta _2^2)}}{\vartheta _3}\tan \bigg (\frac{\sqrt{-(\vartheta _1^2+\vartheta _2^2)}}{2}\sigma \bigg ), \end{aligned}$$

76 $$\begin{aligned} \Theta ^{h(\sigma )}= & {} \frac{\vartheta _1}{\vartheta _3}+\frac{\sqrt{-(\vartheta _1^2+\vartheta _2^2)}}{\vartheta _3}\cot \bigg (\frac{\sqrt{-(\vartheta _1^2+\vartheta _2^2)}}{2}\sigma \bigg ). \end{aligned}$$

*Case: 5* When $\vartheta _1^2-\vartheta _2^2<0$ and $\vartheta _3\ne -\vartheta _2$

77 $$\begin{aligned} \Theta ^{h(\sigma )}= & {} \frac{-\vartheta _1}{\vartheta _3}+\frac{\sqrt{-(\vartheta _1^2-\vartheta _2^2)}}{\vartheta _3}\tan \bigg (\frac{\sqrt{-(\vartheta _1^2-\vartheta _2^2)}}{2}\sigma \bigg ), \end{aligned}$$

78 $$\begin{aligned} \Theta ^{h(\sigma )}= & {} \frac{-\vartheta _1}{\vartheta _3}+\frac{\sqrt{-(\vartheta _1^2-\vartheta _2^2)}}{\vartheta _3}\cot \bigg (\frac{\sqrt{-(\vartheta _1^2-\vartheta _2^2)}}{2}\sigma \bigg ). \end{aligned}$$

*Case: 6* When $\vartheta _1^2-\vartheta _2^2>0$ and $\vartheta _3\ne -\vartheta _2$

79 $$\begin{aligned} \Theta ^{h(\sigma )}= & {} \frac{-\vartheta _1}{\vartheta _3}+\frac{\sqrt{(\vartheta _1^2-\vartheta _2^2)}}{\vartheta _3}\tanh \bigg (\frac{\sqrt{(\vartheta _1^2-\vartheta _2^2)}}{2}\sigma \bigg ), \end{aligned}$$

80 $$\begin{aligned} \Theta ^{h(\sigma )}= & {} \frac{-\vartheta _1}{\vartheta _3}+\frac{\sqrt{(\vartheta _1^2-\vartheta _2^2)}}{\vartheta _3}\coth \bigg (\frac{\sqrt{(\vartheta _1^2-\vartheta _2^2)}}{2}\sigma \bigg ). \end{aligned}$$

*Case: 7* When $\vartheta _2\vartheta _3>0$, $\vartheta _3\ne 0$ and $\vartheta _1=0$

81 $$\begin{aligned} \Theta ^{h(\sigma )}= & {} \sqrt{\frac{-\vartheta _2}{\vartheta _3}}\tanh \bigg (\frac{\sqrt{-\vartheta _2\vartheta _3}}{2}\sigma \bigg ), \end{aligned}$$

82 $$\begin{aligned} \Theta ^{h(\sigma )}= & {} \sqrt{\frac{-\vartheta _2}{\vartheta _3}}\coth \bigg (\frac{\sqrt{-\vartheta _2\vartheta _3}}{2}\sigma \bigg ). \end{aligned}$$

*Case: 8* When $\vartheta _1=0$ and $\vartheta _2=-\vartheta _3$

83 $$\begin{aligned} \Theta ^{h(\sigma )}= & {} \frac{-(1+e^{2\vartheta _2\sigma })\pm \sqrt{2(1+e^{2\vartheta _2\sigma })}}{e^{2\vartheta _2\sigma }-1}\cdot \end{aligned}$$

*Case: 9* When $$\vartheta _1^2=\vartheta _2\vartheta _3$$

84 $$\begin{aligned} \Theta ^{h(\sigma )}=\frac{-\vartheta _2(\vartheta _1\sigma +2)}{\vartheta _1^2\sigma }\cdot \end{aligned}$$

*Case: 10* When $\vartheta _1=k$, $\vartheta _2=2k$ and $\vartheta _3=0$

85 $$\begin{aligned} \Theta ^{h(\sigma )}=e^{\sigma }-1. \end{aligned}$$

*Case: 11* When $\vartheta _1=k$, $\vartheta _3=2k$ and $\vartheta _2=0$

86 $$\begin{aligned} \Theta ^{h(\sigma )}=\frac{e^{\sigma }}{1-e^{\sigma }}\cdot \end{aligned}$$

*Case: 12* When $$2\vartheta _1=\vartheta _2+\vartheta _3$$

87 $$\begin{aligned} \Theta ^{h(\sigma )}=\frac{1+\vartheta _2 e^{\frac{1}{2}(\vartheta _2-\vartheta _3)\sigma }}{ 1+\vartheta _3 e^{\frac{1}{2}(\vartheta _2-\vartheta _3)\sigma }}\cdot \end{aligned}$$

*Case: 13* When $$-2\vartheta _1=\vartheta _2+\vartheta _3$$

88 $$\begin{aligned} \Theta ^{h(\sigma )}=\frac{\vartheta _2+\vartheta _2 e^{\frac{1}{2}(\vartheta _2-\vartheta _3)\sigma }}{ \vartheta _3+\vartheta _3 e^{\frac{1}{2}(\vartheta _2-\vartheta _3)\sigma }}\cdot \end{aligned}$$

*Case: 14* When $$\vartheta _2=0$$

89 $$\begin{aligned} \Theta ^{h(\sigma )}=\frac{\vartheta _1 e^{\vartheta _1\sigma }}{ 1+\frac{\vartheta _3}{2}e^{\vartheta _1\sigma }}\cdot \end{aligned}$$

*Case: 15* When $$\vartheta _2=\vartheta _1=\vartheta _3\ne 0$$

90 $$\begin{aligned} \Theta ^{h(\sigma )}=\frac{-(\vartheta _2\sigma +2)}{\vartheta _2\sigma }\cdot \end{aligned}$$

*Case: 16* When $$\vartheta _2=\vartheta _3$$, $$\vartheta _1=0$$

91 $$\begin{aligned} \Theta ^{h(\sigma )}=\tan \bigg (\frac{\vartheta _2\sigma +c}{2}\bigg ). \end{aligned}$$

*Case: 17* When $$\vartheta _3=0$$

92 $$\begin{aligned} \Theta ^{h(\sigma )}=e^{\vartheta _1\sigma }-\frac{\vartheta _2}{2\vartheta _1}\cdot \end{aligned}$$

*Step: 6* Replacing all the values of $\Theta ^{h(\sigma )}$ from step: 5 into Eq. (67), we get the results for Eq. (64).

## Implementation of new auxiliary equation method

In this context, we analyze the traveling wave profiles for Eq. (1) using Eq. (62) and employing the new auxiliary equation method. The solution can be expressed as

93 $$\begin{aligned} h(\sigma )=b_0+b_1\Theta ^{h(\sigma )}+b_2\Theta ^{2h(\sigma )}. \end{aligned}$$

Inserting Eq. (93) and its derivatives into Eq. (62), and subsequently equating the coefficients of $\Theta ^{h(\sigma )}$, we form a system of algebraic equations. The solution to the resulting equations is provided below

94 $$\begin{aligned} \begin{aligned} b_0=&b_0,~~~~~~b_1=\frac{6\Gamma _1(\alpha ^2-1)}{\gamma (4\Gamma _1\Gamma _3-\Gamma ^2_2)},~~ ~~~b_2=0,~~~~~~~\vartheta _3=\frac{\alpha ^2(\alpha ^2-1)}{4\Gamma _1\Gamma _3-\Gamma ^2_2}\cdot \end{aligned} \end{aligned}$$

Now by utilizing Eq. (94) into Eq. (93), we get

95 $$\begin{aligned} h(\sigma )=b_0+\frac{6\Gamma _1(\alpha ^2-1)}{\gamma (4\Gamma _1\Gamma _3-\Gamma ^2_2)}\Theta ^{2h(\sigma )}. \end{aligned}$$

The traveling wave patterns for Eq. (1) based on the obtained result are

96 $$\begin{aligned} u(z,t)=b_0+\frac{6\Gamma _1(\alpha ^2-1)}{\gamma (4\Gamma _1\Gamma _3-\Gamma ^2_2)}\Theta ^{2h(\sigma )}. \end{aligned}$$

By inserting the solutions specified by Eq. (68) into Eq. (95), the solutions retrieved are;

*Class:1* When $\vartheta ^2_1-\vartheta _2\vartheta _3<0$ and $\vartheta _3\ne 0$

97 $$\begin{aligned} u_1(z,t)= & {} b_0+\frac{6\Gamma _1(\alpha ^2-1)}{\gamma (4\Gamma _1\Gamma _3-\Gamma ^2_2)}\left[ \frac{-\vartheta _1}{\vartheta _3}+\frac{\sqrt{-(\vartheta _1^2-\vartheta _2\vartheta _3)}}{\vartheta _3}\tan \bigg (\frac{\sqrt{-(\vartheta _1 ^2-\vartheta _2\vartheta _3)}}{2}\sigma \bigg )\right] ^2, \end{aligned}$$

98 $$\begin{aligned} u_2(z,t)= & {} b_0+\frac{6\Gamma _1(\alpha ^2-1)}{\gamma (4\Gamma _1\Gamma _3-\Gamma ^2_2)}\left[ \frac{-\vartheta _1}{\vartheta _3}+\frac{\sqrt{-(\vartheta _1^2-\vartheta _2\vartheta _3)}}{\vartheta _3}\cot \bigg (\frac{\sqrt{-(\vartheta _1 ^2-\vartheta _2\vartheta _3)}}{2}\sigma \bigg )\right] ^2. \end{aligned}$$

*Class:2* When $\vartheta _1^2+\vartheta _2\vartheta _3>0$ and $\vartheta _3\ne 0$

99 $$\begin{aligned} u_3(z,t)= & {} b_0+\frac{6\Gamma _1(\alpha ^2-1)}{\gamma (4\Gamma _1\Gamma _3-\Gamma ^2_2)}\left[ \frac{-\vartheta _1}{\vartheta _3}+\frac{\sqrt{(\vartheta _1^2-\vartheta _2\vartheta _3)}}{\vartheta _3}\tanh \bigg (\frac{\sqrt{(\vartheta _1 ^2-\vartheta _2\vartheta _3)}}{2}\sigma \bigg )\right] ^2, \end{aligned}$$

100 $$\begin{aligned} u_4(z,t)= & {} b_0+\frac{6\Gamma _1(\alpha ^2-1)}{\gamma (4\Gamma _1\Gamma _3-\Gamma ^2_2)}\left[ \frac{-\vartheta _1}{\vartheta _3}-\frac{\sqrt{(\vartheta _1^2-\vartheta _2\vartheta _3)}}{\vartheta _3}\coth \bigg (\frac{\sqrt{(\vartheta _1 ^2-\vartheta _2\vartheta _3)}}{2}\sigma \bigg )\right] ^2. \end{aligned}$$

*Class:3* When $\vartheta _1^2+\vartheta _2\vartheta _3>0$ and $\vartheta _3\ne 0$ and $\vartheta _3\ne -\vartheta _2$

101 $$\begin{aligned} u_5(z,t)= & {} b_0+\frac{6\Gamma _1(\alpha ^2-1)}{\gamma (4\Gamma _1\Gamma _3-\Gamma ^2_2)}\left[ \frac{\vartheta _1}{\vartheta _3}+\frac{\sqrt{(\vartheta _1^2+\vartheta _2^2)}}{\vartheta _3}\tanh \bigg (\frac{\sqrt{(\vartheta _1^2+\vartheta _2^2)}}{2}\sigma \bigg )\right] ^2, \end{aligned}$$

102 $$\begin{aligned} u_6(z,t)= & {} b_0+\frac{6\Gamma _1(\alpha ^2-1)}{\gamma (4\Gamma _1\Gamma _3-\Gamma ^2_2)}\left[ \frac{\vartheta _1}{\vartheta _3}+\frac{\sqrt{(\vartheta _1^2+\vartheta _2^2)}}{\vartheta _3}\coth \bigg (\frac{\sqrt{(\vartheta _1^2+\vartheta _2^2)}}{2}\sigma \bigg )\right] ^2. \end{aligned}$$

*Class: 4* When $\vartheta _1^2+\vartheta _2\vartheta _3<0$, $\vartheta _3\ne 0$ and $\vartheta _3\ne -\vartheta _2$

103 $$\begin{aligned} u_7(z,t)= & {} b_0+\frac{6\Gamma _1(\alpha ^2-1)}{\gamma (4\Gamma _1\Gamma _3-\Gamma ^2_2)}\left[ \frac{\vartheta _1}{\vartheta _3}+\frac{\sqrt{-(\vartheta _1^2+\vartheta _2^2)}}{\vartheta _3}\tan \bigg (\frac{\sqrt{-(\vartheta _1^2+\vartheta _2^2)}}{2}\sigma \bigg )\right] ^2, \end{aligned}$$

104 $$\begin{aligned} u_8(z,t)= & {} b_0+\frac{6\Gamma _1(\alpha ^2-1)}{\gamma (4\Gamma _1\Gamma _3-\Gamma ^2_2)}\left[ \frac{\vartheta _1}{\vartheta _3}+\frac{\sqrt{-(\vartheta _1^2+\vartheta _2^2)}}{\vartheta _3}\cot \bigg (\frac{\sqrt{-(\vartheta _1^2+\vartheta _2^2)}}{2}\sigma \bigg )\right] ^2. \end{aligned}$$

*Class: 5* When $\vartheta _1^2-\vartheta _2^2<0$ and $\vartheta _3\ne -\vartheta _2$

105 $$\begin{aligned} u_9(z,t)= & {} b_0+\frac{6\Gamma _1(\alpha ^2-1)}{\gamma (4\Gamma _1\Gamma _3-\Gamma ^2_2)}\left[ \frac{-\vartheta _1}{\vartheta _3}+\frac{\sqrt{-(\vartheta _1^2-\vartheta _2^2)}}{\vartheta _3}\tan \bigg (\frac{\sqrt{-(\vartheta _1^2-\vartheta _2^2)}}{2}\sigma \bigg )\right] ^2, \end{aligned}$$

106 $$\begin{aligned} u_{10}(z,t)= & {} b_0+\frac{6\Gamma _1(\alpha ^2-1)}{\gamma (4\Gamma _1\Gamma _3-\Gamma ^2_2)}\left[ \frac{-\vartheta _1}{\vartheta _3}+\frac{\sqrt{-(\vartheta _1^2-\vartheta _2^2)}}{\vartheta _3}\cot \bigg (\frac{\sqrt{-(\vartheta _1^2-\vartheta _2^2)}}{2}\sigma \bigg )\right] ^2. \end{aligned}$$

*Class: 6* When $\vartheta _1^2-\vartheta _2^2>0$ and $\vartheta _3\ne -\vartheta _2$

107 $$\begin{aligned} u_{11}(z,t)= & {} b_0+\frac{6\Gamma _1(\alpha ^2-1)}{\gamma (4\Gamma _1\Gamma _3-\Gamma ^2_2)}\left[ \frac{-\vartheta _1}{\vartheta _3}+\frac{\sqrt{(\vartheta _1^2-\vartheta _2^2)}}{\vartheta _3}\tanh \bigg (\frac{\sqrt{(\vartheta _1^2-\vartheta _2^2)}}{2}\sigma \bigg )\right] ^2, \end{aligned}$$

108 $$\begin{aligned} u_{12}(z,t)= & {} b_0+\frac{6\Gamma _1(\alpha ^2-1)}{\gamma (4\Gamma _1\Gamma _3-\Gamma ^2_2)}\left[ \frac{-\vartheta _1}{\vartheta _3}+\frac{\sqrt{(\vartheta _1^2-\vartheta _2^2)}}{\vartheta _3}\coth \bigg (\frac{\sqrt{(\vartheta _1^2-\vartheta _2^2)}}{2}\sigma \bigg )\right] ^2. \end{aligned}$$

*Class: 7* When $\vartheta _2\vartheta _3>0$, $\vartheta _3\ne 0$ and $\vartheta _1=0$

109 $$\begin{aligned} u_{13}(z,t)= & {} b_0-\frac{6\Gamma _1(\alpha ^2-1)}{\gamma (4\Gamma _1\Gamma _3-\Gamma ^2_2)}\frac{\vartheta _2}{\vartheta _3}\tanh ^2\bigg (\frac{\sqrt{-\vartheta _2\vartheta _3}}{2}\sigma \bigg ), \end{aligned}$$

110 $$\begin{aligned} u_{14}(z,t)= & {} b_0-\frac{6\Gamma _1(\alpha ^2-1)}{\gamma (4\Gamma _1\Gamma _3-\Gamma ^2_2)}\frac{\vartheta _2}{\vartheta _3}\coth ^2\bigg (\frac{\sqrt{-\vartheta _2\vartheta _3}}{2}\sigma \bigg ). \end{aligned}$$

*Class: 8* When $\vartheta _1=0$ and $\vartheta _2=-\vartheta _3$

111 $$\begin{aligned} u_{15}(z,t)=b_0+\frac{6\Gamma _1(\alpha ^2-1)}{\gamma (4\Gamma _1\Gamma _3-\Gamma ^2_2)}\left[ \frac{-(1+e^{2\vartheta _2\sigma })\pm \sqrt{2(1+e^{2\vartheta _2\sigma })}}{e^{2\vartheta _2\sigma }-1}\right] ^2. \end{aligned}$$

*Class: 9* When $$\vartheta _1^2=\vartheta _2\vartheta _3$$

112 $$\begin{aligned} u_{16}(z,t)=b_0+\frac{6\Gamma _1(\alpha ^2-1)}{\gamma (4\Gamma _1\Gamma _3-\Gamma ^2_2)}\left[ \frac{-\vartheta _2(\vartheta _1\sigma +2)}{\vartheta _1^2\sigma }\right] ^2. \end{aligned}$$

*Class: 10* When $\vartheta _1=k$, $\vartheta _2=2k$ and $\vartheta _3=0$

113 $$\begin{aligned} u_{17}(z,t)=b_0+\frac{6\Gamma _1(\alpha ^2-1)}{\gamma (4\Gamma _1\Gamma _3-\Gamma ^2_2)}(e^{\sigma }-1)^2. \end{aligned}$$

*Class: 11* When $\vartheta _1=k$, $\vartheta _3=2k$ and $\vartheta _2=0$

114 $$\begin{aligned} u_{18}(z,t)=b_0+\frac{6\Gamma _1(\alpha ^2-1)}{\gamma (4\Gamma _1\Gamma _3-\Gamma ^2_2)}\left( \frac{e^{\sigma }}{1-e^{\sigma }}\right) ^2. \end{aligned}$$

*Class: 12* When $$2\vartheta _1=\vartheta _2+\vartheta _3$$

115 $$\begin{aligned} u_{19}(z,t)=b_0+\frac{6\Gamma _1(\alpha ^2-1)}{\gamma (4\Gamma _1\Gamma _3-\Gamma ^2_2)}\left[ \frac{1+\vartheta _2 e^{\frac{1}{2}(\vartheta _2-\vartheta _3)\sigma }}{ 1+\vartheta _3 e^{\frac{1}{2}(\vartheta _2-\vartheta _3)\sigma }}\right] ^2. \end{aligned}$$

*Class: 13* When $$-2\vartheta _1=\vartheta _2+\vartheta _3$$

116 $$\begin{aligned} u_{20}(z,t)=b_0+\frac{6\Gamma _1(\alpha ^2-1)}{\gamma (4\Gamma _1\Gamma _3-\Gamma ^2_2)}\left[ \frac{\vartheta _2+\vartheta _2 e^{\frac{1}{2}(\vartheta _2-\vartheta _3)\sigma }}{ \vartheta _3+\vartheta _3 e^{\frac{1}{2}(\vartheta _2-\vartheta _3)\sigma }}\right] ^2. \end{aligned}$$

*Class: 14* When $$\vartheta _2=0$$

117 $$\begin{aligned} u_{21}(z,t)=b_0+\frac{6\Gamma _1(\alpha ^2-1)}{\gamma (4\Gamma _1\Gamma _3-\Gamma ^2_2)}\left[ \frac{\vartheta _1 e^{\vartheta _1\sigma }}{ 1+\frac{\vartheta _3}{2}e^{\vartheta _1\sigma }}\right] ^2. \end{aligned}$$

*Class: 15* When $$\vartheta _2=\vartheta _1=\vartheta _3\ne 0$$

118 $$\begin{aligned} u_{22}(z,t)=b_0+\frac{6\Gamma _1(\alpha ^2-1)}{\gamma (4\Gamma _1\Gamma _3-\Gamma ^2_2)}\left( \frac{-(\vartheta _2\sigma +2)}{\vartheta _2\sigma }\right) ^2. \end{aligned}$$

*Class: 16* When $$\vartheta _2=\vartheta _3$$, $$\vartheta _1=0$$

119 $$\begin{aligned} u_{23}(z,t)=b_0+\frac{6\Gamma _1(\alpha ^2-1)}{\gamma (4\Gamma _1\Gamma _3-\Gamma ^2_2)}\tan ^2\bigg (\frac{\vartheta _2\sigma +c}{2}\bigg ). \end{aligned}$$

*Class: 17* When $$\vartheta _3=0$$

120 $$\begin{aligned} u_{24}(z,t)=b_0+\frac{6\Gamma _1(\alpha ^2-1)}{\gamma (4\Gamma _1\Gamma _3-\Gamma ^2_2)}\left( e^{\vartheta _1\sigma }-\frac{\vartheta _2}{2\vartheta _1}\right) ^2, \end{aligned}$$

where in all above cases $\sigma = t-z$.

## Physical nature of the obtained solutions

**Figure 1:**
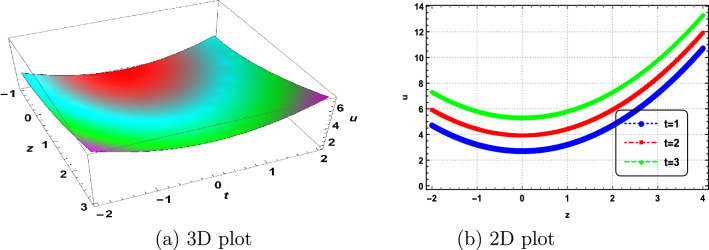
Polynomial nature of displacement in DNA using the invariant solution (51) with $c_1=c_2=1,~\alpha =1,~\gamma =1$ and at t=1,2,3.

**Figure 2:**
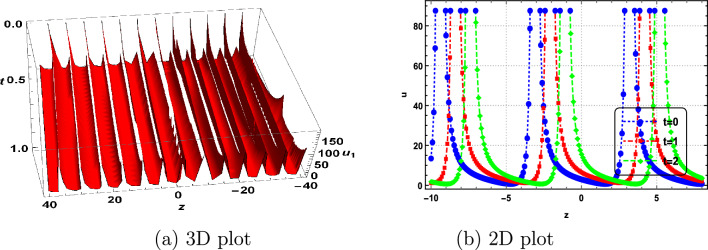
Periodic nature of displacement in DNA using $u_1$ with $\vartheta _1=\vartheta _3=1,~\vartheta _2=2,~b_0=1,~\Gamma _1=\Gamma _2=\Gamma _3=1,~\alpha =\gamma =1$ and at t=0,1,2.

**Figure 3:**
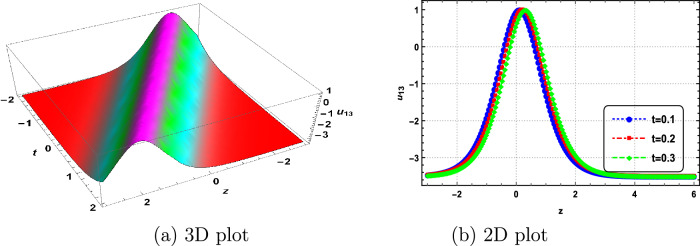
Optical dark soliton nature of displacement in DNA using $u_{13}$ with $\vartheta _1=0,~\vartheta _2=1,~\vartheta _3=1,~b_0=1,~\Gamma _1=\Gamma _2=\Gamma _3=1,~\alpha =2,~\gamma =1$ and at t=0.1,0.2,0.3.

**Figure 4:**
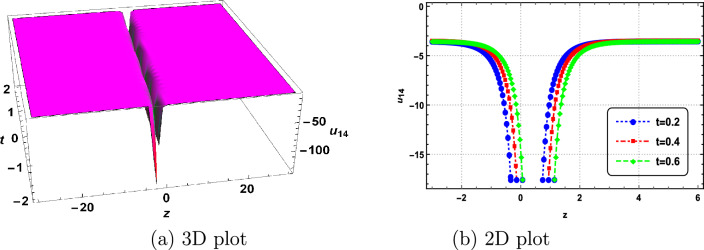
Singular nature of displacement in DNA using $u_{14}$ with $\vartheta _1=0,~\vartheta _2=1,~\vartheta _3=1,~b_0=1,~\Gamma _1=\Gamma _2=\Gamma _3=1,~\alpha =2,~\gamma =1$ and at t=0.2,0.4,0.6.

**Figure 5:**
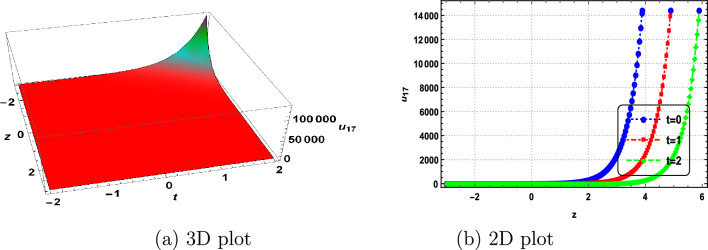
Exponential nature of displacement in DNA using $u_{17}$ with $k=1,~\vartheta _1=1,~\vartheta _2=2,~\vartheta _3=0,~b_0=1,~\Gamma _1=\Gamma _2=\Gamma _3=1,~\alpha =2,~\gamma =1$ and at t=0,1,2.

**Figure 6:**
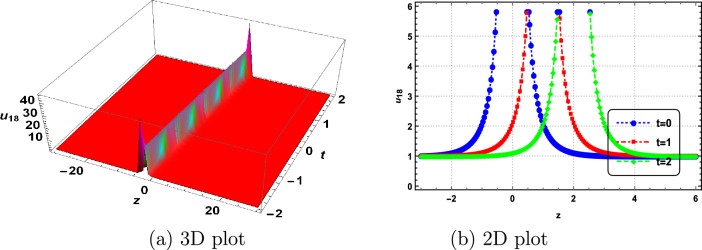
Rational nature of displacement in DNA using $u_{18}$ with $k=1,~\vartheta _1=1,~\vartheta _2=2,~\vartheta _3=0,~b_0=1,~\Gamma _1=\Gamma _2=\Gamma _3=1,~\alpha =2,~\gamma =1$ and at t=0,1,2.

In this section, we delve into the solitonic characteristics of the obtained solutions. Mathematica simulations are employed to identify some recognized structures for the DNA Eq. (1). Figure 1 illustrates the nature of the invariant solution. The periodic solution $u_{1}$ is depicted in Fig. 2. The dynamics of the optical dark soliton solutions $u_{13}$ are explored and presented in Fig. 3. The singular solution $u_{14}$ is also showcased in Fig. 4. The exponential and rational nature of the obtained solutions is illustrated in Figs. 5 and 6, respectively.

## Discussion and conclusions

We have successfully applied the Lie group method to characterize the properties of DNA molecules, specifically addressing the nonlinear dynamics described by Eq. (1). The symmetry algebra for this DNA equation was obtained, and the resulting invariant solutions have been documented. To the best of our knowledge, this study marks the first application of the Lie group method to the dynamics of DNA. The variable *u*(*z*, *t*) in our model represents the difference in longitudinal displacements between the bottom and top strands^1–3^. We have uncovered several intriguing solutions to the nonlinear dynamics of DNA, considering a model consisting of two long elastic homogeneous strands connected by an elastic membrane. This investigation focuses on the longitudinal motions^2^. Therefore, the invariant solutions and the solutions $u_1$ through $u_{24}$ are interpreted as new positions of longitudinal displacements of the strands. Additionally, corresponding simulations are presented in Figs. 1, 2, 3, 4, 5 and 6. Our study contributes novel positions not previously documented in Refs.^1,12–15^.

The interplay of both invariant and waveform solutions governed the longitudinal displacement in DNA, providing insights into the unique characteristics of DNA as a significant real-world challenge. The interactions between DNA and an external microwave field were expressed through various mathematical forms, encompassing rational, exponential, trigonometric, hyperbolic, polynomial, and other functions. Mathematica simulations corroborate these diverse solutions, showcasing longitudinal displacements in DNA as periodic waves, optical dark solitons, singular solutions, exponential forms, and rational forms. This groundbreaking study represents the inaugural application of the Lie group method to explore the interaction of DNA molecules. The findings present novel contributions that have not been reported in the existing literature. The success of this study inspires us to continue utilizing the Lie group method in our future research endeavors.

## Data Availability

All data generated or analyzed during this study are included in this published article.
